# Complete genome sequence of *Vibrio* sp. strain AH4, a close relative of *Vibrio metoecus* isolated from Nile tilapia (*Oreochromis niloticus*)

**DOI:** 10.1128/mra.01219-23

**Published:** 2024-02-22

**Authors:** Ahmed H. Al-Harbi

**Affiliations:** 1 Sustainability and Environment Sector, King Abdulaziz City for Science and Technology, Riyadh, Saudi Arabia; Montana State University, USA

**Keywords:** genome sequence, *Vibrio* sp., tilapia, Illumina, pacific biosciences

## Abstract

Here, I report the complete genome sequence of *Vibrio* sp. strain AH4, which had been isolated from moribund farmed Nile tilapia (*Oreochromis niloticus*). Assessment of the genome sequence of this strain revealed the presence of two linear chromosomes 2,894,109 bp and 1,082,372 bp.

## ANNOUNCEMENT

*Vibrio* species are a diverse group of Gram-negative bacteria ubiquitously present in freshwater and marine aquatic environments (1, 2). Several bacteria species of the *Vibrio* genus are implicated as the etiological agents of disease for a broad range of hosts, including fish (3). Among them, *V. alginolyticus* (4, 5), *V*. *cholerae* (6), and *V. vulnificus* (7–10) have been implicated in tilapia (*Oreochromis* spp.) disease.

*Vibrio* sp. AH4 was originally isolated in 2003 from the kidney of moribund farmed Nile tilapia (*Oreochromis niloticus*) in Saudi Arabia as described previously (11). The *Vibrio* sp. AH4 was maintained at −80°C in tryptone soy broth containing 15% glycerol. For DNA extraction, *Vibrio* sp. AH4 was streaked on tryptone soy agar (Oxoid) and incubated at 30°C for 24 h and a single colony was grown overnight at 30°C in 10 mL tryptone soy broth (Oxoid). Bacterial cells were harvested following centrifugation for 10 min at 10,000 × *g* and were subjected to genomic DNA isolation using a DNeasy blood and tissue kit (Qiagen) following the manufacturer’s instructions. The quantity and quality of the DNA were assessed using a NanoDrop spectrophotometer (Thermo Fisher Scientific, USA) and 1% agarose gel electrophoresis, respectively.

The whole-genomic DNA sequencing was performed using a combination of the Illumina NovaSeq6000 and PacBio RS II platforms. For Illumina, a library was prepared from 1 µg of bacterial genomic DNA fragmented to an average size of ~470 bp using a g-TUBEs devise (Covaris, Woburn, MA, USA). Libraries were generated with NEBNext Ultra II DNA library preparation kit (New England Biolabs, Ipswich, MA, USA), visualized on a 2100 Bioanalyzer (Agilent Technologies, Inc., Santa Clara, CA, USA), and quantified by quantitative PCR (qPCR) with a KAPA library quantification kit (Thermo Fisher Scientific). Paired-end (2 × 150 bp) reads were performed on the NovaSeq 6000 platform (Illumina Inc., USA). Trimmomatic v0.30 was used to trim adapters from the Illumina reads with a sliding window quality cutoff value of Q30 (12). For PacBio sequencing, library was prepared from 5 µg of bacterial genomic DNA sheared using g-TUBEs devise (Covaris, Woburn, MA, USA), size selected to ≥10 kb using the BluePippin system, end repaired and ligated with universal hairpin adapters using the SMRTbell template preparation kit v1.0 (PacBio, Menlo Park, CA, USA) following the manufacturer’s instructions, and sequenced on the RSII platform (PacBio) using P6-C4 chemistry and 240 min movies. The genome was assembled *de novo* with Flye v2.8 (13) and Mecat2 vJAN-2020 software run with default parameters. The assembly was further error corrected and polished using Pilon v1.22 (14). Assembly completeness was assessed using Benchmarking Universal Single-Copy Orthologs (BUSCO) v4.1.4 based on the bacteria_odb10 data set (15). The assembly was annotated with the NCBI Prokaryotic Genome Annotation Pipeline (PGAP) v4.13 (16, 17). Default parameters were used for all software unless otherwise specified.

The assembled complete genome of *Vibrio* sp. AH4 had a total length of 3,979,518 bp, with a G+C content of 47.0%, and it consisted of three contigs (*N*_50_ 14,988 bp) with a total of 3,470 predicted protein-coding genes. The complete genome of *Vibrio* sp. AH4 consists of two linear chromosomes of 2,894,109 bp (GC content of 47.3%) and 1,082,372 bp (GC content of 46.3%). The main genome statistics and features of *Vibrio* sp. AH4 are shown in Table 1. Genome similarity was determined by average nucleotide identity (ANI) using the OrthoANIu algorithm (18), which showed 97.83% and 97.56% identities with the genomes of *V. metoecus* RM-195-2 reference genome (GenBank accession number JAMQXW000000000) and *V. metoecus* OP3H reference genome (GenBank accession number JJMN00000000) respectively.

**TABLE 1:** Genome statistics and features of *Vibrio* sp. strain AH4

Parameter	Value
Accession no.	
Illumina reads accession no.	SRR18101041
PacBio reads accession no.	SRR23799115
Genome assembly accession no.	JALAIV000000000
Sequencing and assembly	
No. Illumina reads	8,555,642
Total no. of Illumina bases	1,121,475,600
No. PacBio reads	336,486
Total no. of PacBio bases	3,713,241,044
Avg genome coverage (x)	931.539
Genome features	
Genome size (bp)	3,979,518
No. of contigs	3
Contig-1 length (chromosome I) (bp)	2,894,109
Contig-4 length (chromosome II) (bp)	1,082,372
Contig-3 length (bp)	3,037
GC content (%)	47
Total no. of genes	3,676
No. of coding sequences	3,535
No. of proteins	3,470
No. of pseudogenes	65
No. of rRNAs	31
No. of tRNAs	106
No. of noncoding RNAs	4
BUSCO results (%)	
Complete	100
Single copy	99.2
Duplicated	0.8
Fragmented	0
Missing	0

## Data Availability

The complete genome sequence of *Vibrio* sp. strain AH4 has been deposited in DDBJ/ENA/GenBank under accession numbers listed in Table 1. The version described in this paper is version JALAIV010000000. The raw sequence reads were deposited in the Sequence Read Archive (SRA) under accession numbers SRR18101041 (Illumina) and SRR17858480 (PacBio). The associated BioProject and BioSample accession numbers are PRJNA809188 and SAMN26149593, respectively.

## AFTER EPUB

*[This article was published on 22 February 2024 with errors in SRA numbers. Table 1 and the Data Availability statement were corrected in the current version, posted on 27 February 2024.]*
